# Genome sequence of phage øR3-PR2, a *Cereibacter sphaeroides* phage isolated from water reservoirs in Puerto Rico

**DOI:** 10.1128/mra.00786-26

**Published:** 2026-09-25

**Authors:** Moisés De Jesús-Cruz, María Fernanda Rojas-Duran, Josue Rodríguez-Ramos, Carlos Rios-Velazquez

**Affiliations:** 1 Microbial Biotechnology and Bioprospecting Laboratory, Biology Department, University of Puerto Rico at Mayagüez, Mayagüez, Puerto Rico, USA; 2 Pacific Northwest National Laboratory, Earth and Biological Science Directorate, Richland, Washington, USA; Portland State University, Portland, Oregon, USA

**Keywords:** *Cereibacter* phage, *Rhodobacter* phage, bacteriophages, lytic bacteriophage, Puerto Rico, water reservoir

## Abstract

A DNA-containing *Cereibacter sphaeroides* phage, designated øR3-PR2, was isolated from water collected from an irrigation canal in Puerto Rico. øR3-PR2 can infect *C. sphaeroides* 7001 and 2.4.1 under aerobic conditions. The φR3-PR2 genome is 52,678 bp long, has a 52.26% G + C content, and encodes 85 predicted protein-coding genes and 1 tRNA-Leu.

## ANNOUNCEMENT

*Cereibacter sphaeroides* (syn. *Rhodobacter sphaeroides*) is a ubiquitous Alphaproteobacteria that grows photoautotrophically, photoheterotrophically, or chemoheterotrophically, depending on the oxygen concentration, carbon source, and light (1). Due to its exceptional metabolic versatility and genomic complexity (consisting of two chromosomes and five endogenous plasmids), *C. sphaeroides* has emerged as a model microorganism and a prominent microbial powerhouse for a wide range of biotechnological and biomedical applications (2).

Phage øR3-PR2 was isolated from water collected from an irrigation canal (currently paved) in Ponce, Puerto Rico (17°58′50.1″ N, 66°36′39.7″ W), using the protocol described by Rojas-Duran et al. (3). Phage amplification (PA) was performed using the mutant strain *Cereibacter sphaeroides* 7001 (4), derived from the wild-type strain *C. sphaeroides* 630 (5). Briefly, 1 mL of filtered (0.2 µm) water sample was inoculated with 100 µL of exponentially growing *C. sphaeroides* 7001 (OD_650_: 0.6) in 10 mL of Luria-Bertani broth (Sigma-Aldrich) and incubated at 32°C for 48 h (constantly shaking). Post-incubation, the samples were centrifuged at 10,000 × *g* for 15 min and filtered through a 0.2 µm membrane. PA was repeated twice using 500 µL of the prior supernatant as the inoculum, and its presence was confirmed via the agar-overlay method (3). One hundred microliters of log-phase *C. sphaeroides* 7001 was mixed with 5 mL of molten soft agar, poured over an agar plate, and allowed to solidify; 5 µL aliquots of filtered phage lysate were spotted onto the overlay, and plates were incubated at 32°C for 24 h. Purification was performed by single-plaque isolation and was amplified as described above. Phage DNA was extracted as described by Sambrook and Russell (6) using Proteinase K and SDS and preliminarily characterized by RFLP analysis (treated with *Cla*I, *Hind*III, and *Eco*RI), showing a range of 33–56 kbp (3).

**Fig 1:**
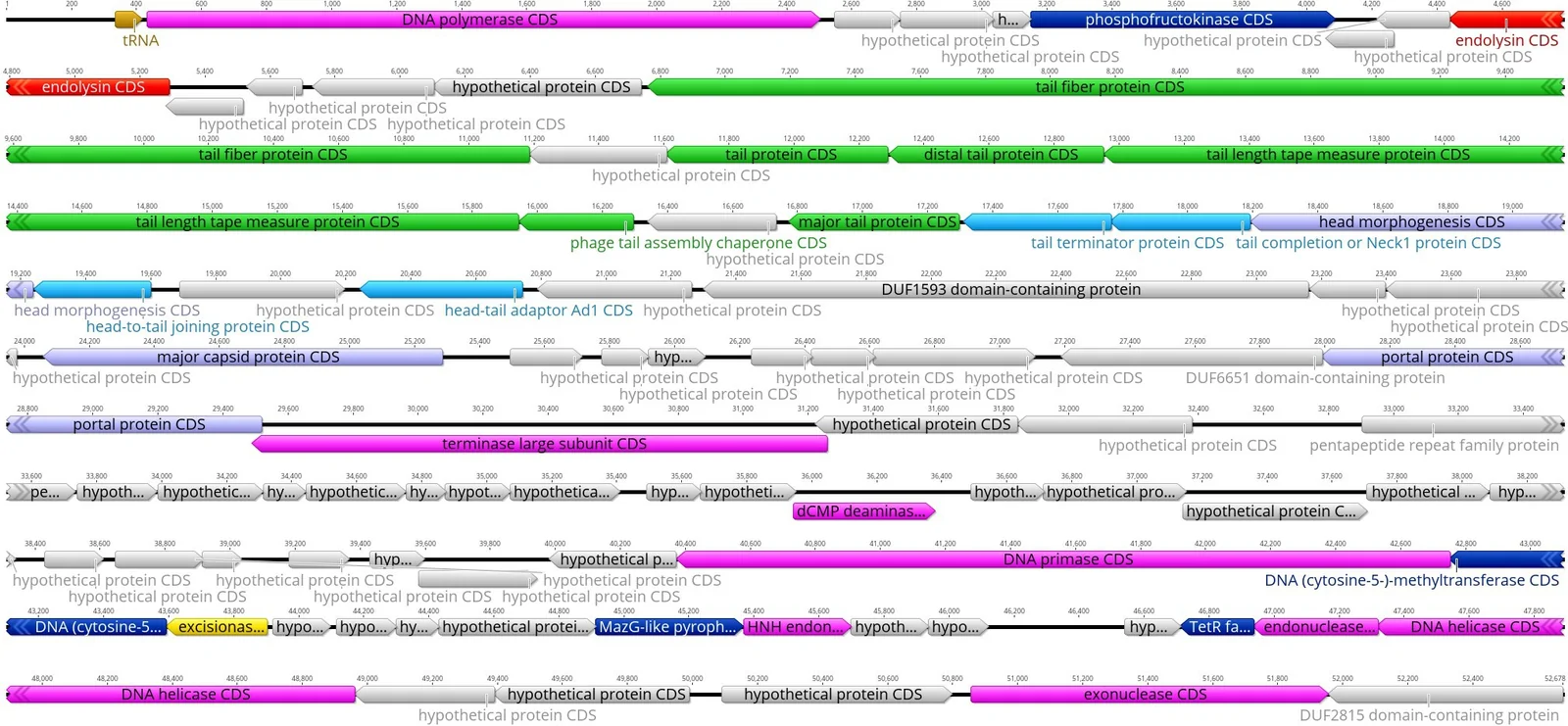
øR3-PR2 genome organization. Arrows indicate predicted genes. Colors indicate different functions, and gray is hypothetical; the total genome molecular weight is 52,678 bp (image created using Geneious v2026.1)

Library preparation and sequencing were done by MR DNA LAB (Shallowater, TX), using the Nextera DNA Sample Preparation Kit (Illumina) and Illumina HiSeq sequencing with a 2 × 150 bp paired-end strategy, yielding 5,039,618 reads (1,289-fold coverage). Reads were processed with BBDuk v38.84 (7) for adapter removal, quality trimming, and filtering. Reads were assembled using the Unicycler v0.4.8 pipeline through the Bacterial and Viral Bioinformatics Resource Center (BV-BRC [8]; https://bv-brc.org, accessed 2 October 2025), generating a sequence of 52,678 bp with a GC content of 52.56%. Genome assembly quality was evaluated using QUAST v5.2.0 (9); coverage was determined using Bowtie2 v2.4.4 (10); and completeness (100%) was verified by CheckV v1.0.1 (11). Coding sequences were predicted using GLIMMER v3.02 (12), while tRNA coding regions were predicted by ARAGORN v19.1.0.0 (13). The function of the called genes was predicted by Pharokka v1.2.0 (14) and BLASTp (15). PhageTerm v1.0.12 (16) analysis detected short direct terminal repeats (90 bp) at approximately 52,631 and 42 and was classified as DTR (short), Type T7. All tools were run with default parameters unless otherwise specified. Analysis of øR3-PR’s genome predicted one tRNA-Leu and 85 protein-coding genes (27 functional predictions), 45 in the forward direction, and 40 in the reverse (Fig. 1). The closest BLASTn hit for phage øR3-PR2 BLASTn closest hit was *Rhizobium* phage RHph_X2_24, a *Caudoviricetes*, with 67.6% pairwise identity (Table 1).

**TABLE 1:** Characterization of *Cereibacter sphaeroides* phage øR3-PR2

Characteristics	Metric
Genome size (bp)	52,678
Contamination	0.0
Coverage (×)	1,289.1
%GC	52.26
Predicted coding sequence	85
tRNA	1 (-Leu)
Closest related phage (GenBank accession no.)	*Rhizobium phage* RHph_X2_24 (MW960031)
Pairwise identity (%)	67.6

## Data Availability

Associated raw reads are available in the SRA database under accession no. SRR38060691, BioProject accession no. PRJNA1452232, and BioSample accession no. SAMN57227366. The whole-genome sequence is available at GenBank with accession no. PZ417139.
